# Drug Exposure of Long-Acting Cabotegravir and Rilpivirine in Older People With Human Immunodeficiency Virus: A Pharmacokinetic Modeling Study

**DOI:** 10.1093/ofid/ofae171

**Published:** 2024-03-21

**Authors:** Sara Bettonte, Mattia Berton, Felix Stader, Manuel Battegay, Catia Marzolini

**Affiliations:** Division of Infectious Diseases and Hospital Epidemiology, Departments of Medicine and Clinical Research, University Hospital Basel, Basel, Switzerland; Faculty of Medicine, University of Basel, Basel, Switzerland; Division of Infectious Diseases and Hospital Epidemiology, Departments of Medicine and Clinical Research, University Hospital Basel, Basel, Switzerland; Faculty of Medicine, University of Basel, Basel, Switzerland; Certara UK Limited, Sheffield, United Kingdom; Division of Infectious Diseases and Hospital Epidemiology, Departments of Medicine and Clinical Research, University Hospital Basel, Basel, Switzerland; Faculty of Medicine, University of Basel, Basel, Switzerland; Division of Infectious Diseases and Hospital Epidemiology, Departments of Medicine and Clinical Research, University Hospital Basel, Basel, Switzerland; Faculty of Medicine, University of Basel, Basel, Switzerland; Department of Molecular and Clinical Pharmacology, University of Liverpool, Liverpool, United Kingdom; Service and Laboratory of Clinical Pharmacology, Department of Laboratory Medicine and Pathology, University Hospital Lausanne and University of Lausanne, Lausanne, Switzerland

**Keywords:** cabotegravir, long acting, older adults, PBPK modeling, rilpivirine

## Abstract

**Background:**

The life expectancy of people with human immunodeficiency virus (PWH) has significantly increased, thanks to combined antiretrovirals with improved potency and tolerability. One further step has been achieved with the development of long-acting (LA) injectable antiretrovirals, which allow for infrequent dosing. However, the pharmacokinetics of LA antiretrovirals has been poorly characterized in older PWH, as they are generally excluded from trials. We performed virtual studies using physiologically based pharmacokinetic (PBPK) modeling to determine the anticipated exposure of LA cabotegravir/rilpivirine in older individuals.

**Methods:**

Our PBPK model was verified against available observed data for LA cabotegravir and rilpivirine. Cohorts of virtual individuals aged 20–50, 50–65, or 65–85 years were generated to simulate the exposure of LA cabotegravir/rilpivirine for each age group. The fold changes in trough concentration (C_min_) and in drug exposure (area under the time-concentration curve [AUC]) were determined for older relative to young individuals.

**Results:**

The verified PBPK models predicted an increase in exposure within the 0.8–1.25 fold range for monthly LA cabotegravir/rilpivirine. The C_min_ and AUC were predicted to be 29% and 26% higher in older compared with young adults for LA cabotegravir administered bimonthly (every 2 months) and 46% and 41% higher for LA rilpivirine bimonthly. The C_min_ and AUC of LA cabotegravir and rilpivirine were predicted to be modestly increased in female compared with male individuals for all age groups.

**Conclusions:**

LA cabotegravir/rilpivirine exposure and trough concentrations are predicted to be higher in older than in young PWH; thus, older adults could have a lower risk to present suboptimal concentrations during the dosing interval.

## Teaser

Monthly and bimonthly long-acting cabotegravir/rilpivirine exposure and trough concentrations are predicted to be higher in older than in young people with human immunodeficiency virus (PWH). Thus, older PWH could have a lower risk of suboptimal concentrations during the dosing interval.

People across the world are living longer. The World Health Organization has estimated that by 2030, 1 in 6 people will be ≥60 years old [1]. A similar trend has been reported for people with human immunodeficiency virus (HIV; PWH) since potent antiretroviral therapy has transformed HIV infection into a chronic disease so that PWH have a near-normal life expectancy [2]. This is notably illustrated by the increasing proportion of older PWH in the Swiss HIV Cohort Study, with approximately 25% of the participants >60 years old at the end of 2022 [3]. Aging is associated with physiological changes that may affect drug pharmacokinetics and pharmacodynamics [4]; however, only limited clinical data are available, given that older adults are generally excluded from clinical trials [5]. This limitation can be overcome with physiologically based pharmacokinetic (PBPK) modeling, a mathematical tool accepted by regulatory agencies [6, 7], which allows simulation of unstudied clinical scenarios such as pharmacokinetics in older individuals [8]. Using PBPK modeling, our group previously demonstrated that the exposure of oral antiretrovirals is increased in older PWH, but to an extent that does not warrant dose adjustment [5, 9].

In recent years, the antiretroviral armamentarium has been expanded by the advent of long-acting (LA) injectable antiretrovirals, for which limited data are available in older PWH. A subanalysis of the ATLAS, FLAIR, and ATLAS-2M studies showed that the tolerability and exposure of LA cabotegravir and rilpivirine were similar in participants aged <50 years and those aged ≥50 years [10, 11]. However, these data may not be reflective of the entire geriatric population, considering that the release of cabotegravir and rilpivirine from the depot can be affected by factors such as blood flow in the muscle or muscle mass, which are reduced with aging [4]. Thus, the main aim of the current study was to use PBPK modeling to simulate virtual clinical trials, in order to investigate the effect of age-related physiological changes on exposure to LA cabotegravir and rilpivirine administered monthly or bimonthly (every 2 months) and to assess whether a dosage adjustment is needed in older PWH. We also explored the effect of sex on the exposure to LA drugs.

## METHODS

We followed 3 steps to simulate the effect of aging on the pharmacokinetics of LA injectable antiretrovirals. First, the drug models for LA cabotegravir and LA rilpivirine were verified against clinical observed data for young (aged <50 years) and middle-aged/older (aged ≥50 years) adults. Second, we simulated the pharmacokinetics of LA cabotegravir and rilpivirine in young (aged 20–50 years), middle-aged (aged 50–65 years), and older (aged 65–85 years) virtual adults (50% female) by applying the same study design used in the ATLAS, FLAIR, and ATLAS-2M trials. Third, the fold change of each relevant pharmacokinetic parameter (ie, peak concentration [C_max_], area under the time-concentration curve [AUC], and trough concentration [C_min_] at week 96) was calculated in middle-aged and older relative to young adults. The fold change was also calculated separately for female and male individuals in the different age groups.

### PBPK Model Verification in Young and Older Individuals

Our in-house perfusion limited whole-body PBPK model, developed in Matlab 2020a [12] was implemented with an intramuscular framework describing the release of the LA drug from the depot [13]. The model was informed by equations describing the physiological changes in a healthy white population aged 20–99 years (body mass index [BMI], 18.5–30 [calculated as weight in kilograms divided by height in meters squared]) [4]. Oral cabotegravir and rilpivirine models were developed and verified in young adults. The verification in older adults was possible only for rilpivirine, as the pharmacokinetics of oral cabotegravir in older adults has not been reported in the literature. To simulate the pharmacokinetics of LA injectable cabotegravir and rilpivirine in different age groups, the PBPK model was also verified against the observed data in young (aged <50 years) and middle-aged/older (aged ≥50 years) adults, using data from the phase III registrational trials (kindly provided by ViiV Healthcare). The drug models were considered verified when the predictions were within 2-fold of the clinically observed data [14, 15]. The drug parameters used to inform the PBPK model have been previously published by our group [16].

### Impact of Age and Sex on the Exposure of LA Cabotegravir and Rilpivirine

The verified PBPK model was used to determine the effect of aging on the pharmacokinetics of oral cabotegravir (30 mg dose once daily) and rilpivirine (25 mg dose once daily) at steady-state in 2 cohorts of 100 virtual individuals (50% female; BMI, 18.5–30) aged 20–50 years (young) or 65–85 years (older). The fold changes in older relative to young individuals were calculated for C_max_, AUC, and C_min_.

**Figure 1.**
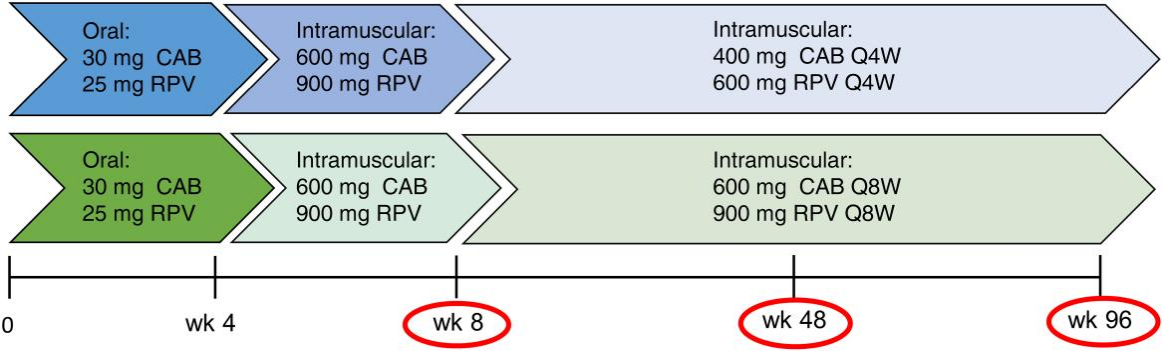
Schematic study design used to determine the exposure of long-acting (LA) cabotegravir (CAB) and rilpivirine (RPV) in young (aged 20–50 years), middle-aged (50–65 years), and older (65–85 years) virtual adults. Physiologically based pharmacokinetic (PBPK) modeling was applied to conduct virtual trials. The design of the phase III registrational studies (ie, FLAIR, ATLAS and ATLAS-2M studies) and of the label dosing recommendations were applied, which consisted of administering an oral lead-in 30 mg for CAB and 25 mg for RPV) for 4 weeks. At week 4, the first intramuscular loading dose was injected (600 mg for CAB and 900 mg for RPV). From week 8 on, the maintenance doses of LA CAB and LA RPV were administered either monthly (every 4 weeks [Q4W]) (400 mg for CAB and 600 mg for RPV) or bimonthly (every 8 weeks [Q8W]) (600 mg for CAB and 900 mg for RPV). Red ovals represent the time points at which the CAB and RPV concentrations were determined at the end of the dosing interval (trough concentrations) for the different age groups.

The PBPK model was subsequently applied to simulate the pharmacokinetics of LA cabotegravir and rilpivirine (after an oral lead-in phase) in young (aged 20–50 years), middle-aged (aged 50–65 years), and older (aged 65–85 years) virtual adults (50% female; BMI, 18.5–30). The design of the simulations described in Figure 1 was aligned with the design of the phase III clinical trials ATLAS/FLAIR and ATLAS-2M and with the label dosing recommendations for LA cabotegravir and rilpivirine [17–19].

At weeks 8, 48, and 96, the percentage of virtual individuals below the protein-adjusted 90% inhibitory concentration (PA-IC_90_) (166 ng/mL [20]) and below 4× PA-IC_90_ (664 ng/mL [20]) for cabotegravir and below the 25th percentile (32 ng/mL [21]) and the minimal concentration for therapeutic response (50 ng/mL [22]) for rilpivirine were calculated. For each age group, the fold changes were calculated for C_min_ and AUC measured at week 96 in middle-aged or older relative to young virtual adults. In addition, the effect of sex was evaluated in young and older virtual adults by generating virtual cohorts with only female or male adults for the different age groups; then the fold changes were calculated for young female relative to young male adults and for older female relative to older male adults.

### Patient Consent Statement

This study does not include factors necessitating patient consent.

## RESULTS

### PBPK Model Verification in Young and Older Individuals

**Figure 2.**
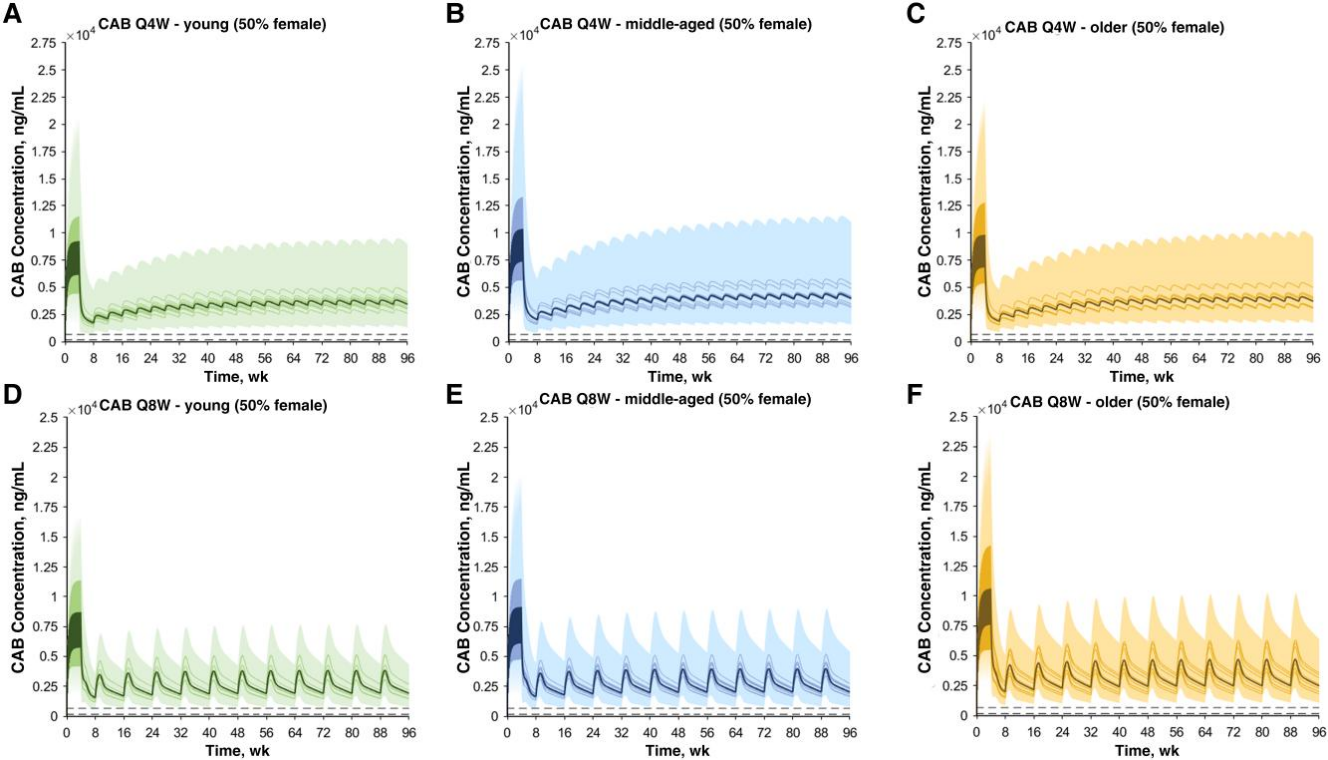
*A–C,* Concentration-time profiles for cabotegravir (CAB) after an oral daily 30-mg dose for 1 month, one 600-mg intramuscular loading dose, and multiple 400-mg maintenance doses administered monthly (every 4 weeks [Q4W]) in young (aged 20–50 years) (*A*), middle-aged (aged 50–65 years) (*B*), and older (aged 65–85 years) (*C*) adults. *D–F,* Concentration-time profiles for CAB after an oral daily 30-mg dose for 1 month, two 600-mg intramuscular loading doses separated by 1 month, and then multiple 600-mg maintenance doses administered bimonthly (every 8 weeks [Q8W]) in young (*D*) middle-aged (*E*), and older (*F*) adults. Solid lines, solid bold lines, and shaded areas represent, respectively, the geometric mean of each virtual trial, the geometric mean of all trials, and the 90% normal range for all virtual individuals. Dashed lines represent the protein-adjusted 90% inhibitory concentration (PA-IC_90_; 166 ng/mL) and the 4× PA-IC_90_ (664 ng/mL) [20].

**Figure 3.**
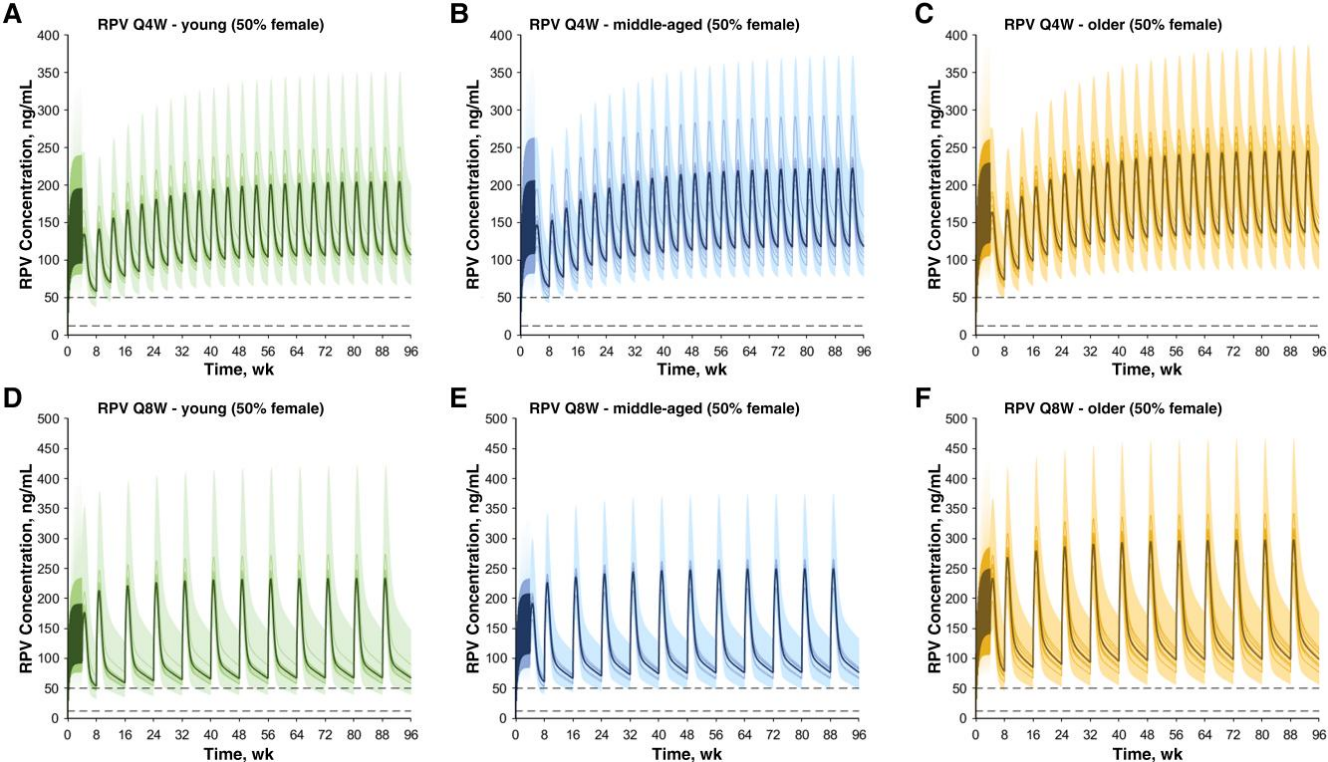
Concentration-time profiles for rilpivirine (RPV) after an oral daily 25 mg dose for 1 month, one 900-mg intramuscular loading dose, and multiple 600-mg maintenance doses administered monthly (every 4 weeks [Q4W]) in young (aged 20–50 years) (*A*), middle-aged (aged 50–65 years) (*B*), and older (aged 65–85 years) (*C*) adults Concentration-time profiles for RPV after an oral daily 2- mg dose for 1 month, two 900-mg intramuscular loading doses separated by 1 month, and then multiple 900-mg maintenance doses administered bimonthly (every 8 weeks [Q8W]) in young (*D*), middle-aged (*E*), and older (*F*) adults. Solid lines, solid bold lines, and shaded areas represent, respectively, the geometric mean of each virtual trial, the geometric mean of all trials, and the 90% normal range for all virtual individuals. Dashed lines represent the fifth percentile (32 ng/mL [21]) and the minimal concentration for therapeutic response (50 ng/mL [22]).

The drug models for cabotegravir and rilpivirine were successfully developed and verified, with all simulations being within 2-fold of clinical observed data after oral and intramuscular administration [13]. The models were also verified against clinical data both for young (aged <50 years) and middle-aged/older (aged ≥50 years) adults (unpublished data provided by ViiV). The simulated cabotegravir and rilpivirine pharmacokinetic profiles for the different age groups are depicted in Figures 2 and 3, respectively.

### Impact of Age and Sex on the Exposure of LA Cabotegravir and Rilpivirine

#### Cabotegravir

After oral administration, cabotegravir C_max_, the AUC to tau (AUC_τ_), and C_min_, were increased by 16%, 20%, and 26%, respectively, in older relative to young adults (Table 1). The full pharmacokinetic profiles after simulation of the ATLAS, FLAIR, and ATLAS-2M studies and the recommended dosing regimens for LA cabotegravir in the different age groups are represented in Figure 2.

**Table 1.** Predicted Pharmacokinetic Values for Oral Cabotegravir and Rilpivirine in Young and Older Individuals (Both 50% Female))

Parameter	Geometric Mean (CV) by Age Group		Ratio Older/Young Adults
	Young (Aged 20–50 y; 50% Female)	Older (Aged 65–85 y; 50% Female)	
Cabotegravir			
C_max_, ng/mL	8526 (49)	9918 (45)	1.16
AUC_τ_, ng ⋅ h/mL	168 700 (57	202 384 (51)	1.20
C_min,_ ng/mL	5493 (67)	6904 (59)	1.26
Rilpivirine			
C_max_, ng/mL	198 (28)	238 (25)	1.20
AUC_τ_, ng ⋅ h/mL	3158 (34)	3946 (28)	1.25
C_min_, ng/mL	98 (43)	132 (33)	1.35

Abbreviations: AUC_τ_, area under the concentration-time curve to tau; C_max_, peak concentration; C_min_, trough concentration; CV, coefficient of variation.

At weeks 8, 48, and 96, none of the virtual individuals in the different age groups receiving LA cabotegravir monthly or bimonthly were below the PA-IC_90_ (166 ng/mL [20]) (Table 2). On the other hand, at week 8, 4% of young, 1% of middle-aged, and 2% of older virtual individuals were below the 4× PA-IC_90_ (664 ng/mL [20]) for the monthly regimen; however, at weeks 48 and 96, none were below this threshold (Table 2). When the maintenance dose of LA cabotegravir was administered bimonthly, at week 8 only 5% of young and middle-aged virtual individuals were below the 4× PA-IC_90_, respectively. At weeks 48 and 96, 3% of young and none of the middle-aged virtual individuals were below the 4× PA-IC_90_, respectively. Importantly, none of the older virtual individuals receiving the maintenance dose bimonthly were below the 4× PA-IC_90_ (Table 2).

**Table 2.** Proportion of Virtual Individuals Below the Protein-Adjusted 90% Inhibitory Concentration (PA-IC_90_) and the 4× PA-IC_90_ Just Before the Next Injection of Long-Acting Cabotegravir, by Age Group and Dosing Regimen^a^

Dosing Regimen	Visit	Sampling Condition	Virtual Individuals With Plasma Concentration <166 ng/mL, %			Virtual Individuals With Plasma Concentration <664 ng/mL, %		
			Young	Middle-Aged	Older	Young	Middle-Aged	Older
Q4W	8 wk	Predose	0	0	0	4	1	2
	48 wk	Predose	0	0	0	0	0	0
	96 wk	Predose	0	0	0	0	0	0
Q8W	8 wk	Predose	0	0	0	5	5	0
	48 wk	Predose	0	0	0	3	0	0
	96 wk	Predose	0	0	0	3	0	0

Abbreviations: Q4W, administered every 4 weeks (monthly); Q8W, administered every 8 weeks (bimonthly).

^a^The PA-IC_90_ is 166 ng/mL [20], and the 4× PA-IC_90_, 664 ng/mL [20]. The age groups were defined as follows: young, aged 20–50 years; middle-aged, aged 50–65 years; and older, aged 65–85 years.

**Figure 4.**
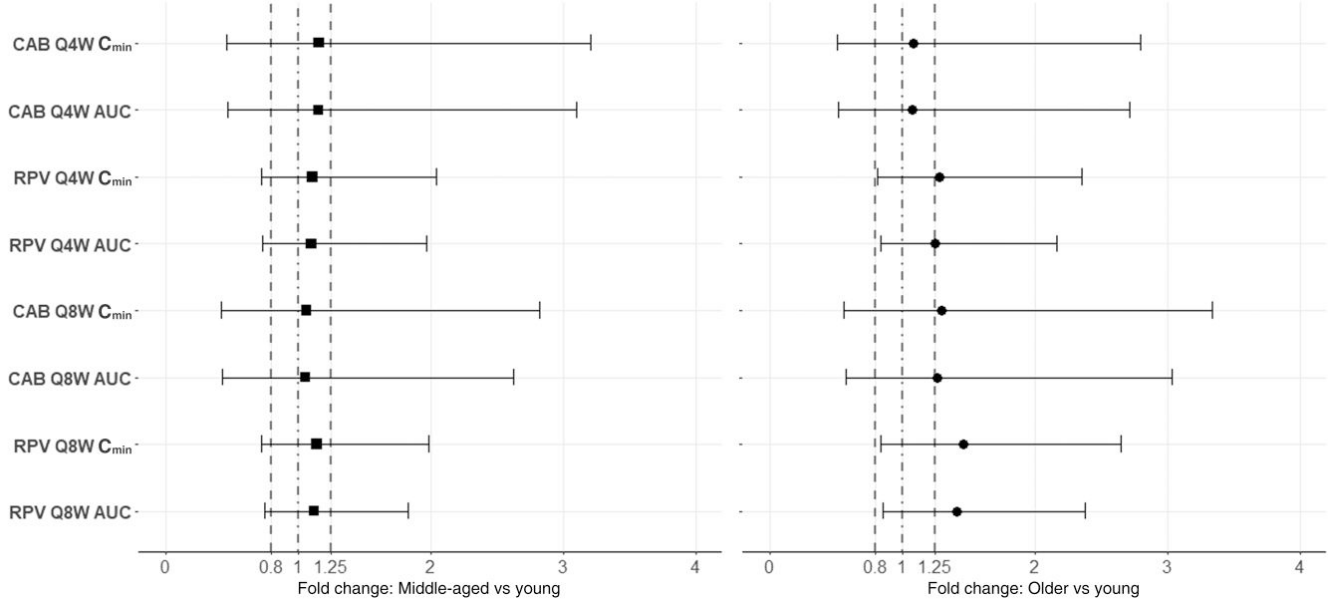
Fold change in exposure (at steady-state administration) in middle-aged (aged 50–65 years) and older (aged 65–85 years) relative to young (aged 20–50 years) adults for long-acting (LA) cabotegravir (CAB) and LA rilpivirine (RPV), administered monthly (every 4 weeks [Q4W]) or bimonthly (every 8 weeks [Q8W]). Results are expressed as geometric mean, fifth percentile, and 95th percentile. Dotted-and-dashed lines represents the unit line; dashed lines, the 0.8–1.25-fold range. Abbreviations: AUC, area under the concentration-time curve; C_min_, trough concentration.

The time to reach steady state concentrations for monthly LA cabotegravir has been previously demonstrated to be 44 weeks [23]; therefore, the fold changes in C_min_ and AUC between middle-aged (50–65 years) or older (65–85 years) relative to young (20–50 years) adults were determined at 96 weeks. Specifically, the change in C_min_ and AUC for middle-aged relative to young adults were within the 0.8–1.25-fold range for both monthly and bimonthly administration (Figure 4). Similarly, the C_min_ and AUC were not significantly increased in older adults receiving the monthly cabotegravir treatment. On the other hand, C_min_ and AUC were increased by 29% and 26%, respectively, in older virtual adults receiving cabotegravir bimonthly (Figure 4).

**Figure 5.**
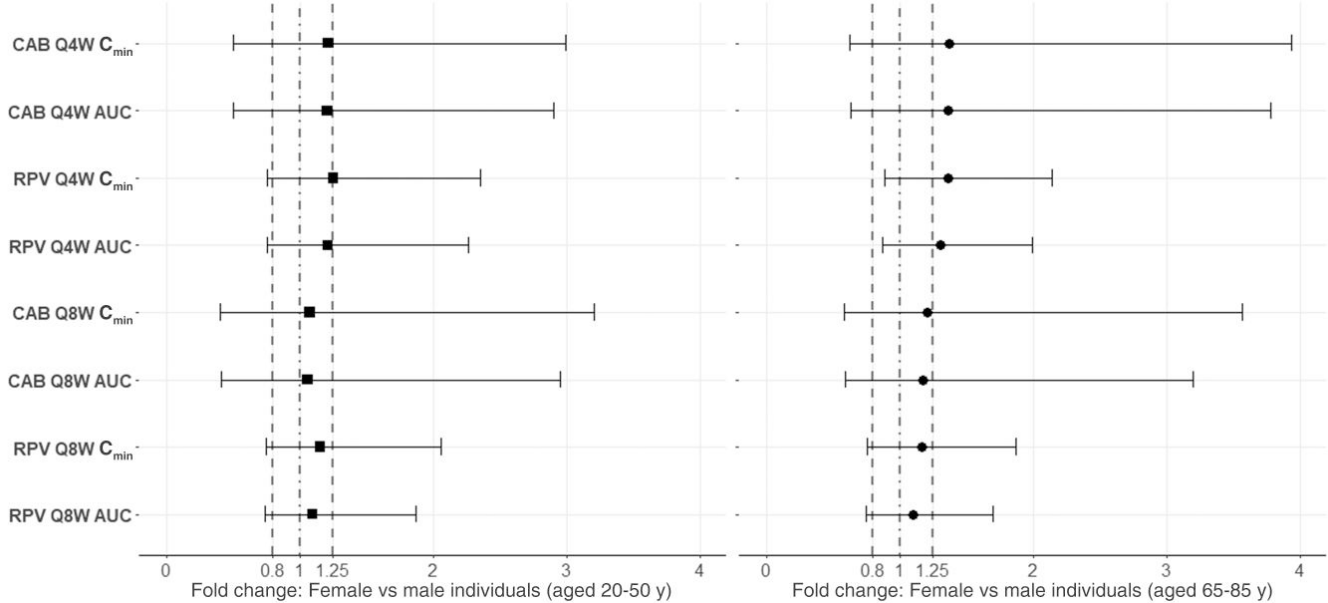
Fold change in exposure (at steady-state administration) in young (aged 20–50 years) female relative to young male individuals and older (aged 65–85 years) female relative to older male adults for long-acting (LA) cabotegravir (CAB) and LA rilpivirine (RPV), administered monthly (every 4 weeks [Q4W]) or bimonthly (every 8 weeks [Q8W]). Results are expressed as geometric mean, fifth percentile, and 95th percentile. Dotted-and-dashed lines represents the unit line; dashed lines, the 0.8–1.25-fold range. Abbreviations: AUC, area under the concentration-time curve; C_min_, trough concentration.

Minimal differences in the pharmacokinetics of LA cabotegravir were found when comparing C_min_ and AUC in young female versus young male individuals for both monthly and bimonthly administration. The C_min_ and AUC were modestly increased in older female compared with older male individuals receiving cabotegravir monthly 37% and 36%, respectively) or bimonthly (21% and 18%); these changes were still within the 0.8–1.25-fold range (Figure 5).

#### Rilpivirine

After oral administration, rilpivirine C_max_, AUC_τ_, and C_min_, were increased by 20%, 25%, and 35%, respectively, in older relative to young adults (Table 1). The full pharmacokinetic profiles after simulation of the ATLAS, FLAIR, and ATLAS-2M studies and the recommended dosing regimens in the different age groups are represented in Figure 3.

At week 8, only 1% of virtual individuals in the young (aged 20–50 years) and middle-aged (aged 50–65 years) groups were below the 25th percentile (32 ng/mL [21]) when receiving LA rilpivirine at a monthly maintenance dose (Table 3). At later time points (ie, weeks 48 and 96), none of the virtual individuals in these age groups were below the 25th percentile. In addition, none of the older virtual individuals receiving the monthly maintenance dose were below the 25th percentile (Table 3). For the bimonthly administration, only 5% of the young virtual individuals were below the 32 ng/mL threshold at week 8; however, none were below the threshold at later time points. Similar results were obtained for middle-aged and older individuals (Table 3).

**Table 3.** Proportion of Virtual Individuals Below the 25th Percentile and the Minimal Concentration for Therapeutic Response Just Before the Next Injection of Long-Acting Rilpivirine, by Age Group and Dosing Regimen^a^

Dosing Regimen	Visit	Sampling Condition	Virtual Individuals With Plasma Concentration <32 ng/mL, %			Virtual Individuals With Plasma Concentration <50 ng/mL, %		
			Young	Middle-Aged	Older	Young	Middle-Aged	Older
Q4W	8 wk	Predose	1	1	0	40	20	9
	48 wk	Predose	0	0	0	0	0	0
	96 wk	Predose	0	0	0	0	0	0
Q8W	8 wk	Predose	5	0	0	48	28	8
	48 wk	Predose	0	0	0	23	8	1
	96 wk	Predose	0	0	0	22	8	0

Abbreviations: Q4W, administered every 4 weeks (monthly); Q8W, administered every 8 weeks (bimonthly).

^a^The 25th percentile was 32 ng/mL [21], and the minimal concentration for therapeutic response was 50 ng/mL [22]. The age groups were defined as follows: young, aged 20–50 years; middle-aged, aged 50–65 years; and older, aged 65–85 years.

On the other hand, 40% of young, 20% of middle-aged, and 9% of older virtual individuals were below the minimal efficacy concentration (50 ng/mL [22]) for the monthly rilpivirine maintenance administration. However, at weeks 48 and 96, none of the virtual individuals were below this threshold (Table 3). When considering the bimonthly maintenance dosing, 48%, 28%, and 8% of the virtual individuals in the young, middle-aged, and older groups, respectively, were below 50 ng/mL at week 8. At week 48, 23% of young, 8% of middle-aged, and 1% of older individuals were below the 50 ng/mL threshold. Similar percentages were calculated at week 96 (Table 3).

The time to reach steady-state concentrations for monthly LA rilpivirine has been previously demonstrated to be mostly 48 weeks [23]; therefore, as for LA cabotegravir, the fold changes in C_min_ and AUC between middle-aged (aged 50–65 years) or older (aged 65–85 years) relative to young (aged 20–50 years) adults were evaluated at 96 weeks. Specifically, the changes in C_min_ and AUC for the middle-aged relative to young individuals were within the 0.8–1.25-fold range for both monthly and bimonthly administration (Figure 4). On the other hand, C_min_ and AUC were increased by 28% and 25%, respectively, in older virtual individuals receiving rilpivirine monthly, and by 46% and 41% in those receiving rilpivirine bimonthly (Figure 4).

Minimal differences in the pharmacokinetics of LA rilpivirine were found when comparing C_min_ and AUC in young female versus young male individuals for both monthly and bimonthly administration (Figure 5). Similarly, in older female individuals receiving LA rilpivirine bimonthly the increases in C_min_ and AUC were not clinically relevant (Figure 5). In addition, the C_min_ and AUC were increased by 36% and 30%, respectively, in older female compared with older male individuals receiving LA rilpivirine monthly, which is not considered clinically relevant (Figure 5).

## DISCUSSION

Our group previously demonstrated that the exposure of oral antiretrovirals is increased in older adults; however, to an extent that does not require dose adjustment [5, 9]. The physiological and lifestyle changes occurring with aging (ie, reduced physical activity, muscle mass, and muscle blood flow) also have the potential to alter the pharmacokinetics of LA intramuscular cabotegravir and rilpivirine. Due to limited clinical data in older PWH, we conducted virtual pharmacokinetic trials using PBPK modeling to evaluate the effect of aging on oral (for the lead-in phase) and intramuscular cabotegravir and rilpivirine.

Our models showed that aging caused a modest increase in the exposure of oral cabotegravir (AUC increased by 20%) and rilpivirine (AUC increased by 25%). Similarly, aging was predicted to modestly affect the pharmacokinetics of intramuscular LA cabotegravir and rilpivirine after monthly or bimonthly administration. For LA cabotegravir, the increase in exposure with aging resulted in concentrations above the PA-IC_90_ (166 ng/mL [20]) and the 4× PA-IC_90_ (664 ng/mL [20]) thresholds over the dosing interval at week 96 for all older virtual adults (aged 65–85 years). The same was predicted for LA rilpivirine, for which none of the older virtual adults were below the 25th percentile (32 ng/mL [21]) or the minimal concentration for therapeutic response (50 ng/mL [22]), unlike findings in young or middle-aged adults. These results suggest that older PWH (aged 65–85 years) could have a lower risk of suboptimal drug exposure during the dosing interval. Our findings also indicate that no dose adjustment is needed a priori for LA cabotegravir and rilpivirine in older PWH in the absence of major comorbid conditions, as their exposure is predicted to be increased by approximately 30% (bimonthly administration). This increase is still below the limit associated with an enhanced risk of QTc interval prolongation for rilpivirine (500 ng/mL) [24].

The phase III FLAIR, ATLAS, and ATLAS-2M studies showed that the percentages of confirmed virological failure (defined as 2 consecutive measurements of ≥200 copies/mL) were comparable in those aged <50 years (young adults) or ≥50 years (older adults) [10, 11]. The confirmed virological failure rates at weeks 48 and 96 in older PWH were indeed reported to be 1.1% and 1.7%, respectively, for monthly and 0% and 1.1%, respectively, for bimonthly administration. Based on these data, the authors concluded that the efficacy of LA cabotegravir/rilpivirine and the occurrence of confirmed virological failure were similar in young and older PWH, although very few participants were >65 years old [10, 11]. Our findings further support these clinical observations, as we demonstrated that older adults—and, to some lesser extent, middle-aged adults—have higher LA cabotegravir/rilpivirine exposures than young adults. As secondary aim, we could also demonstrate that the exposure to LA cabotegravir and rilpivirine is higher in female than in male individuals at steady state, irrespectively of age. Our finding is consistent with observed clinical data showing higher cabotegravir exposure at steady state in female compared with male individuals [25, 26]. Furthermore, it is in line with our previous work compiling existing clinical data and demonstrating higher exposure in female than in male individuals [27].

Several limitations should be acknowledged. First, the population physiology used to inform the PBPK model described the physiological changes in a fit older adult population without major comorbid conditions [4]. However, it should be noted that the effect of frailty on drug pharmacokinetics has been hardly studied. The design of a clinical trial in frail subjects is challenging due to the wide heterogeneity, leading to high variability in pharmacokinetics and pharmacodynamics [28]. Specifically, frail older people are characterized by reduced gastric motility and liver metabolism, sarcopenia, (ie, loss of skeletal muscle mass and strength as a result of low-grade inflammation [29, 30]), decreased lean body mass, lower albumin levels and reduced renal function [28]. These changes are anticipated to further increase the exposure of LA injectables in frail PWH compared with fit older PWH. Thus, further studies will also need to determine the impact of frailty on LA antiretroviral response. Second, the pharmacokinetics of oral cabotegravir has not been characterized in older PWH during clinical trials; however, our simulations showed an increase in AUC comparable to that reported in older individuals for the structural analogue integrase inhibitor dolutegravir (AUC increased by 16%) [5].

Third, no supportive data are available in the literature regarding differences in LA cabotegravir and rilpivirine exposures in older female and male individuals. Finally, the PBPK model simulated the ideal clinical scenario in which all virtual individuals received the injection in the muscle, whereas real-world data have shown injection site variability [31]. Thus, our model does not reproduce the variability reported in real-world studies [25, 32, 33], which was not always explained by differences in BMI [34]. As matter of fact, young and older individuals with similar BMIs may have different tissue distributions [35] which may increase the risk of receiving the injection in the adipose tissue rather than the muscle. Moreover, the variability could be related to changes in blood flow [33, 34]. Thus, further data are needed to investigate the source of variability in the pharmacokinetics of LA injectables together with the related drug response in both young and older PWH.

In conclusion, the exposures to LA cabotegravir and rilpivirine were predicted to be increased in older PWH, but to an extent that does not warrant a dose adjustment in the absence of major comorbid conditions. This finding is reassuring, as it suggests that older PWH could have a lower risk of presenting suboptimal concentrations during the dosing interval. Moreover, the exposures to LA cabotegravir and rilpivirine at steady state were shown to be higher in young or older female compared with young or older male individuals, but to an extent that does not require dose adjustment. Our simulation results should be interpreted with caution in frail individuals, as further clinical studies are needed to investigate the impact of aging and frailty on LA drug exposures and efficacy.

## Notes

***Acknowledgments.*** The authors are grateful to ViiV Healthcare for providing long-acting cabotegravir and rilpivirine concentrations and the related participants’ demographic data for verification of the physiologically based pharmacokinetic models.

***Author contributions.*** S. B. collected the data, performed the simulations, analyzed the data, and wrote the first draft of the manuscript. M. Berton contributed to the data analysis and writing of the manuscript. F. S. provided modeling input and supervised the data analysis. M. Battegay provided clinical input. C. M. designed the study, provided clinical input, supervised the data analysis, and obtained funding. All authors contributed to the critical review and approval of the manuscript.

***Data availability.*** The data that support the findings of this study are available on request to the corresponding author.

***Financial support.*** This work was supported by the Swiss National Foundation (grant 188504).
